# Prevalence and factors of COVID-19 vaccine refusal among solid cancer patients in China: an application of the health belief model

**DOI:** 10.3389/fpubh.2023.1236376

**Published:** 2023-08-03

**Authors:** Zhaomin Xie, Joseph Tak-Fai Lau, Yuanke Liang, Qiaolei Ouyang, Junjia Chen, Si Lin, Kaitao Yao, Xuanyin Hu, Haoyu Lin, Yanqiu Yu, De Zeng

**Affiliations:** ^1^School of Public Health, Shantou University, Shantou, China; ^2^Department of Medical Oncology, Cancer Hospital of Shantou University Medical College, Shantou, China; ^3^Guangdong Provincial Key Laboratory for Breast Cancer Diagnosis and Treatment, Cancer Hospital of Shantou University Medical College, Shantou, Guangdong, China; ^4^School of Mental Health, Wenzhou Medical University, Wenzhou, China; ^5^Zhejiang Provincial Clinical Research Center for Mental Disorders, The Affiliated Wenzhou Kangning Hospital, Wenzhou Medical University, Wenzhou, China; ^6^School of Public Health, Zhejiang University, Hangzhou, China; ^7^Centre for Health Behaviours Research, Jockey Club School of Public Health and Primary Care, The Chinese University of Hong Kong, Hong Kong, China; ^8^Department of Thyroid and Breast Surgery, Clinical Research Center, The First Affiliated Hospital of Shantou University Medical College (SUMC), Shantou, China; ^9^Shantou University Medical College, Shantou, China; ^10^Department of Preventive Medicine and Health Education, School of Public Health, Fudan University, Shanghai, China

**Keywords:** cancer patients, health belief model, COVID-19, vaccine refusals, vaccine

## Abstract

**Introduction:**

It is essential to protect cancer patients from contracting COVID-19 through vaccination. A majority of cancer patients are recommended by international health authorities to take up the vaccines. COVID-19 vaccine refusal among cancer patients during the pandemic period is under-researched. This study investigated factors of vaccine refusal based on the Health Belief Model (HBM).

**Methods:**

A cross-sectional study was conducted among female breast cancer patients, male/female thyroid cancer patients, and gynecological cancer patients in Shantou, China from April to August 2022 (*n* = 1,115). Multinomial logistic regression analysis adjusted for socio-demographics was conducted to test factors of COVID-19. Adjusted odds ratios of the two models comparing vaccine refusal vs. “vaccine non-refusal” and vaccine refusal vs. ever-vaccination were derived and presented.

**Results:**

Of all the participants, the prevalence of vaccine refusal, “vaccine non-refusal,” and ever-vaccination was 25.9, 22.2, and 51.8%, respectively. In both multinomial logistic regression models, significant factors of vaccine refusal included socio-demographics (age, education level, employment status, monthly household income, cancer type, duration since cancer diagnosis, current treatment status) and some vaccine-related HBM (perceived benefits, perceived barriers, cue to action, and self-efficacy). Perceived severity of COVID-19 was significant only in the vaccine refusal vs. ever-vaccination model. In neither model, perceived susceptibility to contract COVID-19 was statistically significant.

**Conclusion:**

About ¼ of the participants expressed vaccine refusal. Interventions are warranted. Future longitudinal studies are needed to verify this study’s findings. Pilot interventions should also be launched to test effectiveness of interventions modifying the significant HBM factors found in this study.

## Introduction

The COVID-19 pandemic has created global severe disease and financial burdens (1). The health consequences of COVID-19 infection are particularly serious in some diseased groups (2, 3). Vaccination is known to be effective in controlling the pandemic (4). It was estimated that COVID-19 vaccines have averted 19.8 million deaths in the first year since their rollout (5). In particular, COVID-19 is a threat to cancer patients who are more vulnerable to severe harms and deaths resulting from COVID-19 than the general population (6–9). One study reported that among COVID-19 patients, those suffering from cancer showed a higher fatality rate and a higher risk of severe complications related to COVID-19 than their counterparts (7). Nationwide data collected in China also showed that cancer patients have higher prevalence of COVID-19 infection than the general population (10). The threat of COVID-19 on cancer patients prevails. Thus, COVID-19 vaccination in this population is highly warranted.

In general, perceived safety and perceived efficacy of COVID-19 vaccination are strong determinants of vaccine hesitancy (11, 12). A study showed that tolerance of COVID-19 vaccination among cancer patients receiving systematic treatments was indistinguishable from that of the general population (13). Another study found a similar incidence of adverse events related to COVID-19 vaccination between cancer patients and non-patients (14). A prospective multicenter study revealed that the predominant adverse events of COVID-19 vaccination among cancer patients were mild and self-resolving reactions of injection site pain and anorexia, suggesting that COVID-19 vaccines among cancer patients are safe in general (15). In addition, a large-scale cohort study showed that COVID-19 vaccines could reduce COVID-19 infection in cancer patients (16). A randomized clinical trial found that cancer patients aged 80 years and older were still able to develop serological responses 1 month after receiving COVID-19 vaccines (17). Thus, there is no evidence that cancer patients should refrain from vaccination.

In contrast, global health authorities, including those specializing in oncology, have recommended that cancer patients should be given a high priority to receive COVID-19 vaccines (18–22). The American Society of Clinical Oncology and the Vaccination Advisory Committee of the National Comprehensive Cancer Network recommend that cancer patients, including those who are active or receiving cancer treatment, should be prioritized for the COVID-19 vaccine, while patients who have recently received hematopoietic stem cell transplantation or chimeric antigen receptor T cell therapy should postpone vaccination for at least 3 months (23, 24). The European Society of Cancer Sciences also recommends vaccination among patients who have finished treatments or who are in stable conditions, while there are reservations for those under active cancer treatments (19). According to the “Chinese Expert Consensus on Issues Related to the Protection, Treatment and Management of Patients with Solid Tumors during COVID-19 (2022)” (25), patients undergoing treatments of surgery, radiotherapy, chemotherapy and immunotherapy and those showing allergy to vaccine components should suspend or refrain from vaccination, while those on endocrine therapy and targeted therapy can receive vaccines immediately after doctor’s evaluations. Thus, vaccination is recommended by the majority of cancer patients. High vaccination rates have been observed among cancer patients in countries such as Germany (95%) (26), Japan (75%) (27), and Canada (86.8%) (28). In many countries, the prevalence of COVID-19 vaccination in cancer patients was, however, low or relatively low. For instance, it was 41.8% in Bosnia and Herzegovina (29), 50.5% in Tunisia (30), 66.0% in Mexico (31), and 19.5% in Korea (32). Despite the official recommendations, the prevalence only ranged from 12.6 to 58.8% among cancer patients in China (33–39).

Inclination toward COVID-19 vaccination has been studied in various dimensions, including willingness to take up the vaccines (40), vaccine hesitancy (41), and vaccine refusal (42). Many people had held an initial ‘wait-and-see’ attitude in response to the uncertainties regarding COVID-19 vaccination had eventually taken up the vaccines (43, 44). Vaccine refusal differs from vaccine hesitancy (45). Instead of considering whether to take up COVID-19 vaccination, people may hold a firm stance on refusing vaccination under any circumstances (46). A better understanding of vaccine refusal has particular importance as its prevalence is critical in determining the eventual vaccination coverage and a high coverage is required to achieve community or herd immunity (47). Notably, a dearth of studies has investigated factors of COVID-19 vaccination behavior and inclinations among cancer patients. Such information may facilitate the design of effective health promotion programs. Similar to other populations, cancer patients’ concerns about the safety and efficacy of COVID-19 vaccination were negatively associated with vaccine acceptance (13, 40). Other factors of low vaccine acceptance included female gender, older age, disease status (32), fear of interaction between vaccination and treatment effect (30), and a lack of knowledge about vaccination (33).

Furthermore, it is warranted to understand theory-based factors of COVID-19 vaccination among cancer patients. The Health Belief Model (HBM) (48) was used as the theoretical framework in the present study. It postulates that perceived severity and perceived susceptibility of the disease (COVID-19 in this case) and perceived benefits, perceived barriers, self-efficacy, and cue to action related to the health-related behavior are determinants of the behavior (46, 47, 49–52) [COVID-19 vaccination in this case (53, 54)]. Such HBM factors can be modified through health promotion and interventions (55). The HBM constructs were able to predict vaccination behaviors in the populations (56), such as human papillomavirus vaccines (57), influenza vaccines (58), and COVID-19 vaccines (59, 60) Notably, cancer patients’ HBM cognitions related to COVID-19 and COVID-19 vaccines may differ from those of the general population (13, 42), and are understudied.

The present study investigated (a) the prevalence of COVID-19 vaccination behavior (i.e., ever-vaccination) and two types of vaccination inclinations (“vaccine non-refusal” and vaccine refusal) among four groups of cancer patients in China who had not taken up COVID-19 vaccination prior to their cancer diagnosis (male and female thyroid cancer patients, female breast cancer patients and gynecological cancer patients), and (b) the levels of related HBM factors. The associations between the HBM factors and vaccine refusal vs. ever-vaccination/vaccine refusal vs. “vaccine non-refusal” were tested. In this study, the “vaccine non-refusal” group referred to those who had neither taken up vaccination nor definitely refusing to take up COVID-19 vaccination in the future. i.e., they planning or thinking about whether to be vaccinated. Our literature search could not locate studies investigating vaccine refusal or applying the HBM to understand COVID-19 vaccination behavior/inclinations among cancer patients.

## Methods

### Study design and participants

A cross-sectional study was conducted among cancer patients in four major hospitals from April to August 2022 in Shantou city, China, which is located in Guangdong province in southern China and has a population of 5.7 million people. The four conveniently selected hospitals (the Affiliated Cancer Hospital of Shantou University, the First and the Second Affiliated Hospitals of Shantou University, and the Shantou Central Hospital) provided medical care to about 80% of the city’s cancer patients. The inclusion criteria included: (1) Chinese residents aged ≥18 years, (2) primary diagnosis of breast cancer (females only) or thyroid cancer (males and females) or gynecological cancer (“gynecological cancer” refers to cancers that specifically originate in the female reproductive organs, including the cervix, ovaries, uterus, fallopian tubes, vulva, and vagina), and (3) provision of written informed consent. The exclusion criteria included: (1) at least one dose of COVID-19 vaccination taken up prior to cancer diagnosis, (2) multiple primary cancer diagnoses, (3) terminal cancer conditions, (4) currently or recently under cancer treatment of palliative care, chemotherapy, radiotherapy, surgery and immunotherapy, (5) physically unfit for vaccination, and (6) cognitive impairment.

Two modes of recruitment were implemented. The first one involved on-site recruitments conducted in the selected hospitals with the assistance of the clinical staff. Cancer patients visiting the hospitals for follow-up consultations were screened according to the inclusion/exclusion criteria. The nurses referred eligible prospective participants who were fit to take up the vaccines to contact the onsite research staff. The trained fieldworkers then explained the objectives, content, and the anonymous nature of the survey to the participants, and guaranteed to them that refusal to participate in the survey or termination at any time point would not cause any negative consequences, nor would affect their rights to use any services. In a private setting and with written informed consent, the participants self-administered an anonymous structured questionnaire which took about 10 min to complete. Upon completion, the investigator collected the questionnaires and conducted onsite quality check and sought clarifications if necessary. Second, a telephone survey was conducted by trained interviewers to further recruit eligible cancer patients who had not visited the hospitals during the study period, using patient records as the sampling frame. With similar inclusion/exclusion criteria, briefing, and consent procedures, the interviewers obtained verbal informed consent and administered the telephone survey using an identical questionnaire. No incentives were given to the participants. This study was approved by the Ethics Committee of the Cancer Hospital of Shantou University Medical College, Shantou, China (Reference Number 2022034).

The initial sample size was 1,303, among which 188 (14.43%) were excluded due to (a) poor quality (e.g., taking less than 1.5 min to fill out the questionnaire; *n* = 24), (b) COVID-19 vaccination prior to cancer diagnosis (*n* = 100), and (c) primary cancer diagnoses other than the breast cancer, thyroid cancer, and gynecological cancers (*n* = 64). The final effective sample size was 1,115, of whom 412 and 703 were recruited on site and via the telephone survey, respectively.

### Measures

The expert panel based the development of the questionnaire on a comprehensive literature review of COVID-19 vaccination studies conducted specifically among cancer patients. The literature review encompassed a wide range of research articles, studies, and publications that provided valuable insights into the vaccination experiences, beliefs, and factors influencing vaccination choices in this specific population (61, 62). While established measures and questionnaires exist for assessing the HBM components, the decision to devise new questions was made to ensure the cultural relevance and appropriateness of the items for the population of cancer patients in this study. By developing new questions through the expert panel, we aimed to capture the nuances and context-specific factors that may influence vaccination decision-making among cancer patients in our specific setting. A pilot survey was conducted among 10 cancer patients to assess clarity, readability, and length of the draft questionnaire. With their feedback, the panel finalized the questionnaire.

#### Background characteristics

(a) Socio-demographic characteristics included age, gender, monthly income, marital status, education level, number of family members, and employment status. (b) Body Mass Index [BMI] (kg/ m^2^) was calculated by using calibrated machines to measure weight and height (underweight: <18.5 kg/m^2^, normal: 18.5–23.9 kg/cm^2^, overweight: 24.0–27.9 kg/cm^2^, and obese: ≥28.0 kg/cm^2^). (c) Cancer-related variables included (i) cancer type (female breast cancer, male thyroid cancer, female thyroid patients, and gynecological cancer patients), (ii) current treatment status [e.g., endocrine therapy, targeted therapy, and treatments that would not affect the suitability of COVID-19 vaccination according to the some official guideline (25)] (yes/no), and (iii) duration since cancer diagnosis.

#### COVID-19 vaccination behavior/inclination status

Participants were classified into three categories: (a) the ever-vaccination group (those who had taken up COVID-19 vaccines after their cancer diagnosis), (b) the “vaccine non-refusal group,” i.e., those who were planning or thinking about whether to take up the vaccines instead of definitely refusing any COVID-19 vaccination in the future, and (c) the vaccine refusal group (those who decided that they would definitely not take up the COVID-19 vaccines in the future). In addition, groups (b) and (c) were asked about the reasons for not having taken up the vaccines in a close-ended multi-choice question.

#### HBM variables

A number of summative scales (see Supplementary Table S1) were constructed in this study, including (a) the 2-item Perceived Susceptibility Scale (Cronbach’s alpha = 0.73; range = 0–8; reversed scores), the 3-item Perceived Severity Scale (Cronbach’s alpha = 0.89; range = 0–12), the 3-item Perceived Benefits Scale (Cronbach’s alpha = 0.81; range = 0–12), the 3-item Perceived Barriers Scale (Cronbach’s alpha = 0.81; range = 0–12), the 1-item Self-Efficacy Scale (range = 0–4). Such scales were assessed by 5-point Likert scales (0 = strongly disagree to 4 = strongly agree). High scores represent higher levels of these constructs. “Cue to action” is a concept within the HBM that refers to a trigger or stimulus that prompts an individual to take action toward a specific health behavior. In our research, cue to Action of COVID-19 vaccination was assessed by asking whether the participants had been suggested to take up COVID-19 vaccination by their family members, their friends, doctors/nurses, and members of community or village committees, respectively (yes/no). An indicator variable was formed by counting the number of affirmative responses [0 (the reference group), 1, 2 or above].

## Statistical analysis

As the categorical dependent variable of COVID-19 vaccination status had three groups (i.e., ever-vaccination, “vaccine non-refusal” and vaccine refusal), multinomial logistic regression analysis was used to generate two models comparing vaccine refusal vs. ever-vaccination and vaccine refusal vs. “vaccine non-refusal” (63). As the focus was put on vaccine refusal, the results of the third comparison of ‘vaccine non-refusal’ vs. ever-vaccination was presented in Supplementary Table S2 instead of in the main text. Univariable and adjusted multinomial logistic regression analyses were conducted to test the individual factors (i.e., the HBM variables) of vaccine refusal, both in the absence and presence of adjustment for the significant background factors, respectively. Univariate odds ratios (ORu), adjusted odds ratios (ORa), and their corresponding 95% confidence intervals (CIs) were reported. Data analyses was conducted by using SPSS 25.0. Two-sided *p* < 0.05 was considered statistically significant.

## Results

### Descriptive statistics

The results are shown in Table 1. The majority (95.2%) of the participants was currently married; 19.9% had received an education level of college or above; 43.5% had had a full-time job; 35.2% had had five or more family members; 56.7% had had a monthly household income of >6,000 RMB (about 880 USD). About half were aged 50 or below (52%). The BMI data showed that 4.7 and 37.6% were underweight and overweight/obese, respectively. About half (52%) were currently under cancer treatments that should not affect vaccination (e.g., endocrine therapy, oral targeted drugs, Chinese traditional medicine); 21.5% had had disease duration >5 years since cancer diagnosis. Regarding the independent variables, the mean scores of the HBM variables were 2.2 for perceived susceptibility (SD = 1.6, range = 0–8), 6.3 for perceived severity (SD = 2.3, range = 0–12), 8.5 (for perceived benefit SD = 2.1, range = 0–12), 5.5 for perceived barrier (SD = 2.8, range = 0–12), and 2.7 for self-efficacy (SD = 1.2, range = 0–12). Regarding the cue to action indicator, 57.5, 20.2, 11.8, 4.8, and 5.5% of the participants had received suggestions to take up vaccination from 0, 1, 2, 3, and 4 sources (family members: 21.7%; good friends: 11.6%; doctors/nurses: neighborhood community committee members: 21.0%. see Supplementary Table S1).

**Table 1:** Descriptive statistics (*n* = 1,115).

Variables	Count	Proportion (%)
**Vaccination behavior/inclination status**		
Vaccine refusal	289	25.9
“Vaccine non-refusal”	248	22.2
Ever-vaccination	578	51.8
**Age group (years)**		
>50	535	48.0
≤50	580	52.0
**BMI (kg/m**^**2**^**)**		
<18.5	52	4.7
<23.9	644	57.8
24 ~ 27.9	359	32.2
≥28	60	5.4
**Currently marital status**		
Not married	54	4.8
Married	1,061	95.2
**Educational level**		
Below college level	893	80.1
College or above	222	19.9
**Employment status**		
Full-time job	485	43.5
Housewife	333	29.9
Retiree	169	15.2
Unemployed	102	9.1
Others	26	2.3
**Number of family members**		
≥5	393	35.2
3 ~ 4	640	57.4
0 ~ 2	82	7.4
**Monthly household income (RMB)**		
≤6,000	483	43.3
>6,000	632	56.7
**Cancer type**		
Thyroid cancer (male)	41	3.7
Thyroid cancer (female)	159	14.3
Breast cancer (female)	553	49.6
Gynecological cancer	362	32.5
**Currently under treatment (other than palliative care, surgery, radiation, chemotherapy)**		
Yes	580	52.0
No	535	48.0
**Duration since cancer diagnosis (year)**		
<1	87	7.8
1–3	544	48.8
3–5	244	21.9
>5	240	21.5
**Cue to action indicator (number of types of suggestion)**		
0	641	57.5
1	226	20.2
2	132	11.8
3	54	4.8
4	62	5.5

BMI, Body Mass Index; RMB, Renminbi.

#### Prevalence of ever-vaccination, “vaccine non-refusal,” and vaccine refusal

The prevalence of ever-vaccination, “vaccine non-refusal,” and vaccine refusal was 51.8, 22.2, and 25.9%, respectively. In Table 2, cancer type (*p* < 0.001) but not age (*p* = 0.062) was significantly associated with vaccination behavior/inclination. The prevalence of ever-vaccination was presented in an ascending order of 33.3, 65.5, 76.1, 87.8% for the female breast cancer group, the gynecological cancer group, the female thyroid cancer group, and the male thyroid cancer group, respectively. In reverse, the prevalence of vaccine refusal was 33.6, 22.9, 10.7, 7.3% in the four corresponding groups, respectively (*p* < 0.001).

**Table 2:** COVID-19 vaccination behavior/inclination by age group and cancer type (*n* = 1,115).

Variables	Vaccination behavior/inclination status (%)			Chi-square	*p* value
	Vaccine refusal	“Vaccine non-refusal”	Ever-vaccination		
All	289 (25.9)	248 (22.2)	578 (51.8)		
**Age group (years)**					
>50	155 (29.0)	119 (22.2)	261 (48.8)	5.54	0.062
≤50	134 (23.1)	129 (22.2)	317 (54.7)		
**Cancer type**					
Thyroid cancer (male)	3 (7.3)	2 (4.9)	36 (87.8)	170.77	<0.001
Thyroid cancer (female)	17 (10.7)	21 (13.2)	121 (76.1)		
Breast cancer (female)	186 (33.6)	183 (33.1)	184 (33.3)		
Gynecological cancer	83 (22.9)	42 (11.6)	237 (65.5)		

### Reasons for not taking up COVID-19 vaccination after cancer diagnosis

As shown in Table 3, Of the 537 unvaccinated participants, over 10% mentioned the following reasons for not having taken up COVID-19 vaccination: perceived poor health (51.6%), unknown side effects of vaccination in cancer patients (36.5%), fear about potential interactions between COVID-19 vaccines and cancer treatments (35.0%), recommendations against vaccination given by healthcare workers (26.8%), perception that cancer was more serious than COVID-19 (21.2%), perceived stronger side effects in cancer patients than the general population (17.9%), low perceived risk of COVID-19 infection (17.7%), unsupportive attitude among family members or friends (12.9%), and logistics issues (11.4%).

**Table 3:** Reasons for hesitancy in accepting COVID-19 vaccine (*n* = 537).

Items	Factors	Count	Proportion (%)
A	I think I'm in poor health to get vaccinated	277	51.6
B	The effect of the vaccine on cancer patients is unknown	196	36.5
C	Fear of interaction of COVID-19 vaccine with the active anticancer treatment	188	35.0
D	Healthcare workers do not recommend	144	26.8
E	COVID-19 is less serious than cancer	114	21.2
F	The side effects of vaccination are higher in cancer patients	96	17.9
G	I think the risk of contracting COVID-19 is very low	95	17.7
H	Family, friends, etc. do not support	69	12.9
I	It is inconvenient and difficult to vaccinate COVID-19 vaccine	61	11.4
J	I don’t think the COVID-19 vaccines work very well.	55	10.2
K	The vaccine is unsafe.	48	8.9
L	Other	35	6.5
M	None of the surrounding cancer patients have been vaccinated	22	4.1

### Background factors of COVID-19 vaccine refusal

As shown in Table 4, those aged >50 years (reference group: ≤50), having attained an education level lower than college (reference group: college or above), being currently unemployed (reference group: others), having breast cancer diagnosis (female) having disease duration since cancer diagnosis for <1 year or 1–3 years (reference group ≥5 years), and being currently under treatment were more likely than others to report vaccine refusal than ever-vaccination and only having thyroid cancer diagnosis (both male and female) (reference group: gynecological cancer) was more likely than others to report ever-vaccination than vaccine refusal. Similarly, those having a monthly income ≤6,000 RMB (reference group: >6,000), breast cancer (female) or thyroid cancer diagnosis (female) (reference group: gynecological cancer), duration since cancer diagnosis of <1 year (reference: ≥5 years) were more likely than others to belong to the vaccine refusal group than to the “vaccine non-refusal” group.

**Table 4:** Background factors of vaccine refusal (Univariable multinomial logistic regression).

Variables	Vaccine refusal vs. ever-vaccination	Vaccine refusal vs. “vaccine non-refusal”
	ORu (95% CI)	ORu (95% CI)
**Age (years)**		
>50	1.41 (1.06, 1.87)*	1.25 (0.89, 1.76)
≤50	Ref = 1.0	Ref = 1.0
**BMI (kg/m**^**2**^**)**		
<18.5	0.78 (0.32, 1.94)	0.71 (0.24, 2.05)
<23.9	0.89 (0.48, 1.66)	0.86 (0.40, 1.83)
24 ~ 27.9	0.87 (0.46, 1.65)	0.97 (0.44, 2.13)
≥28	Ref = 1.0	Ref = 1.0
**Current marital status**		
Not married	1.1 (0.53, 2.25)	0.49 (0.24, 1.03)
Married	Ref = 1.0	Ref = 1.0
**Educational level**		
Below college level	1.52 (1.05, 2.18)*	0.97 (0.61, 1.53)
College or above	Ref = 1.0	Ref = 1.0
**Employment status**		
Full-time job	0.69 (0.25, 1.86)	1.23 (0.40, 3.80)
Housewife	1.49 (0.55, 4.05)	2.12 (0.68, 6.60)
Retiree	1.06 (0.37, 2.99)	0.84 (0.26, 2.69)
Unemployed	3.25 (1.10, 9.64)*	1.23 (0.38, 4.00)
Other	Ref = 1.0	Ref = 1.0
**Number of family members**		
≥5	1.69 (0.92, 3.1)	1.38 (0.69, 2.78)
3 ~ 4	1.18 (0.65, 2.12)	1.42 (0.71, 2.82)
0 ~ 2	Ref = 1.0	Ref = 1.0
**Monthly household income (RMB)**		
≤6,000	0.84 (0.63, 1.12)	0.68 (0.49, 0.97)*
>6,000	Ref = 1.0	Ref = 1.0
**Cancer type**		
Thyroid cancer (male)	0.24 (0.07, 0.79)*	0.76 (0.12, 4.72)
Thyroid cancer (female)	0.40 (0.23, 0.71)*	0.41 (0.20, 0.86)*
Breast cancer (female)	2.89 (2.09, 3.99)***	0.51 (0.34, 0.79)*
Gynecological cancer	Ref = 1.0	Ref = 1.0
**Duration since cancer diagnosis (year)**		
<1	4.34 (2.27, 8.28)***	0.49 (0.25, 0.99)*
1 ~ 3	3.27 (2.20, 4.85)***	0.80 (0.47, 1.36)
3 ~ 5	1.25 (0.78, 2.00)	0.91 (0.48, 1.74)
>5	Ref = 1.0	Ref = 1.0
**Current treatment status**		
Yes	1.78 (1.34, 2.37)***	0.78 (0.55, 1.10)
No	Ref = 1.0	Ref = 1.0

BMI, Body Mass Index; RMB, Renminbi; ORu, Univariate odds ratio; CI, Confidence interval; *p < 0.05; ***p < 0.001.

### Adjusted analysis for the HBM factors of vaccine refusal

As shown in Table 5, the adjusted models showed that those participants with stronger perceived severity of COVID-19 (ORa: 1.31, 95% CI: 1.06–1.61), stronger perceived barrier of COVID-19 vaccination (ORa: 24.84, 95% CI: 16.29–37.88), lower perceived benefit of COVID-19 vaccination (ORa: 0.11, 95% CI: 0.08–0.16), and lower self-efficacy regarding COVID-19 vaccination (ORa: 0.15, 95% CI: 0.12–0.19) were more likely than those ever-vaccinated to show vaccine refusal. Reversely, those exposed to stronger cues to action [reference: no suggestion given; one source (ORa: 0.07, 95% CI: 0.04–0.12), 2–4 sources (ORa: 0.02, 95% CI: 0.01–0.04)] were less likely than others exhibit vaccine refusal than those ever-vaccinated. The same factors were found for the model of refusal vs. ‘vaccine non-refusal’ except that perceived severity of COVID-19 was non-significant in this but not the former comparison.

**Table 5:** Adjusted associations between the HBM Variables and COVID-19 vaccine refusal.

HBM Variables	Vaccine refusal vs. ever-vaccination	Vaccine refusal vs. “Vaccine non-refusal”
	ORa (95% CI)	ORa (95% CI)
Perceived susceptibility	0.91 (0.74, 1.13)	0.9 (0.71, 1.15)
Perceived severity	1.31 (1.06, 1.61)*	1.06 (0.83, 1.37)
Perceived benefits	0.11 (0.08, 0.16)***	0.37 (0.27, 0.5)***
Perceived barriers	24.84 (16.29, 37.88)***	1.82 (1.36, 2.45)***
**Cue to Action Indicator (number of sources of suggestion)**		
2–4	0.02 (0.01, 0.04)***	0.34 (0.15, 0.82)*
1	0.07 (0.04, 0.12)***	0.56 (0.32, 0.95)*
0	Ref	Ref
Self-efficacy	0.15 (0.12, 0.19)***	0.47 (0.39, 0.56)***

These models adjusted for age, current marital status, education level, employment status, monthly household income, cancer type, duration since cancer diagnosis, current treatment status. RMB, Renminbi; HBM, Health Belief Model; ORa, adjusted odds ratio; CI, Confidence interval; *p < 0.05; ***p < 0.001.

## Discussion

This study observed that only about half of the sampled cancer patients had taken up at least one dose of the COVID-19 vaccines at the survey time (April to August 2022). Some socio-demographic factors (e.g., cancer type) of vaccine refusal were identified. It is interesting that the HBM factors related to the vaccines (perceived benefits, perceived barriers, cue to action and self-efficacy) were significantly associated with vaccine refusal in both models (vs. ever-vaccination and vs. “vaccine non-refusal”). Yet, perceived susceptibility of COVID-19 was not significant in both models while perceived severity of COVID-19 was significant in the model of vaccine refusal vs. ever-vaccination but not in that of vaccine refusal vs. “vaccine non-refusal.”

Notably, the prevalence of COVID-19 vaccination in this study (51.8%) was lower than the concurrent prevalence of vaccination in the general population in Shantou (>90%) where the study was conducted during the concurrent time period (64). It was also much lower than that observed among cancer patients in countries such as Canada, Germany, and Japan (26, 27, 65, 66). The vaccination coverage in the sampled cancer patients was hence sub-optimal and probably inadequate to protect the cancer patients against COVID-19 infection. Completion of two doses of vaccination is required for effective protection against COVID-19; such prevalence must even be lower than that of 1-dose vaccination reported hereby. Health promotion is greatly warranted.

Unvaccinated cancer patients commonly mentioned perceived poor health (51.6%), unknown side effects of vaccination in cancer patients (36.5%), fear about potential interactions between COVID-19 vaccines and cancer treatments (35.0%) as reasons for not taking up the vaccines. Such findings corroborate other COVID-19 vaccination studies targeting cancer patients. A Korean study found a positive correlation between patients’ health and acceptance of COVID-19 vaccines (67). A Tunisian study showed that 15.5% of cancer patients refused to take up COVID-19 vaccination as they believed that the vaccines might affect therapeutic effects (67). Among the Italian cancer patients who refused to take up the COVID-19 vaccines, 48.1% worried about adverse reactions and 26.7% were afraid of potential interactions between COVID-19 vaccines and cancer treatments (42, 67). Notably, COVID-19 is still a health threat to cancer patients, presently and in the future. Thus, COVID-19 vaccination is warranted. The aforementioned reasons are specific to cancer patients and are implicative for tailored interventions. Hence, concerns about side effects and interaction effects between vaccines and cancer treatments need to be clarified by health professionals to cancer patients who are suitable for vaccination. In particular, the local and international official expert recommendations for vaccination among cancer patients (25) should be widely disseminated to cancer patients and stakeholders (e.g., family members and health professionals) to facilitate informed vaccination decisions.

Furthermore, about one quarter of the patients did not vaccinate because they had been advised against vaccination by some health professionals, even that these patients seemed eligible according to our inclusion/exclusion criteria and conversations/observations. It is uncertain they were aware of the aforementioned guidelines. Several previous studies have demonstrated that doctors’ recommendation was a significant predictor of vaccination behaviors (27, 40, 67). The government should hence ensure both dissemination of those official guidelines about the exact advices about COVID-19 vaccination given to cancer patients to all health professionals that doctors would give such recommendations to oncology patients accordingly. About one fifth of the participants did not vaccinate as they believed that COVID-19 was less severe than COVID-19. Such patients might have under-estimated the severity of COVID-19 for cancer patients and should be informed about the consequences of COVID-19 among cancer patients.

Some significant socio-demographic factors of vaccine refusal have been identified in this study, including older age, lower educational level, and unemployment status, which was consistent with previous surveys (32, 40). It is plausible that those of older age had had stronger concerns over the safety of COVID-19 vaccination (52) as relevant news and social media often mentioned vaccine-related deaths in order people (68). Similarly, those of lower socio-economic status (e.g., lower educational level and unemployment status) might be older in age and/or less informed about the relatively high efficacy and low side effect of COVID-19 vaccination among cancer patients (69), leading to potential vaccine refusal. Health promotion should target such socio-demographic groups.

Three cancer-related background factors of vaccine refusal were identified. First, thyroid cancer patients were less likely and female breast cancer patients were more likely than gynecological cancer patients to indicate vaccine refusal. The primary site of cancer patients may affect cancer patients’ vaccination behavior, as it involves different symptoms and treatment plans. However, some previous studies also showed that the primary cancer site did not affect patients’ willingness to take up COVID-19 vaccines (32, 70). Such inconsistent results should be examined in future studies. Second, disease duration was inversely associated with vaccine refusal, corroborating a previous multi-center study (71). The sampled cancer patients have relatively good prognosis. The sampled patients might regard a longer duration since diagnosis as a better chance of recovery (72); such patients might hence worry less about the potential side effects of vaccination on their course of the cancer disease. Third, those undergoing treatments other than chemotherapy, radiotherapy and surgery (predominantly endocrinal therapy) were more likely than those not undergoing any treatment to refuse COVID-19 vaccination, possibly due to worries about potential interactions between vaccines and those current treatments. Again, clear, consistent, and transparent information about the suitability of COVID-19 vaccination should be provided to cancer patients, especially those of specific cancer types and undergoing treatments.

The HBM has been partially supported by the data. It is interesting that all the four constructs related to COVID-19 vaccines (perceived benefit, perceived barriers, cue to action, and self-efficacy) were consistently associated with vaccine refusal and in the expected directions. Although there is a dearth of studies applying the HBM to look at vaccine refusal among cancer patients, this study’s findings are consistent with those regarding COVID-19 vaccination behavior (36), acceptance (73), and hesitancy (54) in general populations. Thus, health promotion strategies for reducing vaccine refusal may need to modify such perceptions. A remark for such programs is that the contents should be closely tailored to cancer patients.

The number of sources of cue to action showed a strong negative association with vaccine refusal. As only 21.7, 26.2, 11.6, and 21.0% of the participants had received suggestions for COVID-19 vaccination from family members, health professionals, neighborhood community members, and friends, respectively, there are rooms for improvement. Social influences on COVID-19 vaccination hesitancy are well known (74). As vaccine hesitancy was also common in the general population (75) and family members are influential in determining health-related behaviors of cancer patients (44), family members’ objection for COVID-19 vaccination among diseased people is expected to be common and impactful (76–78). It seems that successful vaccine promotion campaigns targeting cancer patients need to involve patients’ family members (31, 40). Neighborhood community committee is a special feature in China. It maintains close contacts with the community residents to help dealing with their daily problems and disease prevention (79). It has played an important role in promotion of COVID-19 testing, prevention, and vaccination (33). As the majority of the participants have not received supportive suggestions about COVID-19 vaccination from such committees, improvements could be made. Furthermore, despite significance and potential effectiveness, about 73.8% has not received any suggestions regarding COVID-19 vaccination from health professionals, while 26% had even been advised against vaccination by health professionals. Again, training and improvements are warranted. Health professional need to become facilitators instead of barriers of cancer patients’ COVID-19 vaccination.

While the vaccine-related perceptions were significantly associated with vaccine refusal, such was untrue regarding perceptions toward COVID-19. Unlike other studies conducted in some general populations (80), perceive susceptibility was not associated with vaccine refusal. It is plausible that the study was conducted at a time when prevalence of COVID-19 was very low in Shantou. During the study period, indeed, zero cases were detected per day in Shantou (81). In addition, cancer patients were more likely than others to take up preventive measures such as staying at home (82). Such measures might have lowered their perceived susceptibility. Perceived severity was significant when comparing vaccine refusal vs. ever-vaccination but not vs. “vaccine non-refusal,” although in general, this construct was a significant factor of COVID-19 vaccination behavior/acceptance (80). It suggests that promotion of perceived severity of COVID-19 might not be effective to shift the cognitions among the unvaccinated cancer patients from refusal to ‘non-refusal’. This observation may be particularly true when COVID-19 symptoms become milder in the later phases of the pandemic. A theoretical contribution of the findings is that some HBM constructs might have different applications to COVID-19 vaccination in cancer patients vs. general populations.

This study has some limitations. First, the selection of cancer types focused on female breast cancer, gynecological tumors, and thyroid cancer due to their high prevalence and relatively good prognosis. The sample was hence unrepresentative of all cancer types. Such selection may overrepresented female cancer patients. Consequently, this study did not use sex as an independent background factor of vaccine refusal. Relatedly, this study excluded male breast cancer patients due to the small number in the sample (*n* = 2). Second, this study was cross-sectional in design, making it unable to determine the causal or temporal relationships between the independent variables and vaccine refusal. Third, this study classified the patients into the three categories of ever-vaccination, “vaccine non-refusal,” and vaccine refusal. Notably, “vaccine non-refusal” was a relatively heterogeneous group including those of different stages of change (83) regarding vaccination (e.g., contemplation and preparation stages). As few previous studies have applied the HBM to investigating COVID-19 vaccination among cancer patients, the instruments were created in this study. As COVID-19 vaccination may be seen as a socially desirable behavior (19), reporting bias may have occurred. Finally, some variables affecting COVID-19 vaccination in cancer patients may not have been included in this study, The impact of these factors on vaccination choices in cancer patients and their potential implications for public health interventions should be further investigated.

In conclusion, this study reported relatively high prevalence of vaccine refusal against COVID-19 vaccination and relatively low prevalence of first-dose vaccination behavior among the four groups of cancer patients in a Chinese city. It was based on a relatively large sample size. The associations between the HBM constructs (those related to health beliefs related to the vaccines) and vaccine refusal (vs. ever-vaccination and vs. vaccine refusal) were partially supported by the data. Factors distinguishing vaccine refusal vs. ever-vaccination and vaccine refusal vs. “vaccine non-refusal” were largely similar. Future confirmation of the above findings in longitudinal studies are needed, possibly with an extension to other cancer groups. Pilot randomized control trials are also warranted to modify the significant HBM factors to reduce vaccine refusal in cancer patients.

## Data availability statement

The raw data supporting the conclusions of this article will be made available by the authors, without undue reservation.

## Ethics statement

This study was approved by the Ethics Committee of the Cancer Hospital of Shantou University Medical College, Shantou, China (Reference Number 2022034). Participants provided their written informed consent to participate in this study.

## Author contributions

ZX, DZ, HL, and YL contributed to the data acquisition, analysis, interpretation, and drafting the manuscript. JL contributed to conceptualization, questionnaire design, major revision, and finalization of the paper. YY contributed to data analysis, major revision, and finalization of the paper. ZX, JL, and YY verified the data. QO, JC, SL, KY, and XH contributed to data collection. All authors made substantial contribution to the conception of the work and had full access to all the data in the study, revised the work critically, gave final approval of the manuscript submitted for publication, and agreed to be responsible for all aspects of work.

## Funding

This work was supported by Guangdong Basic and Applied Basic Research Foundation (No. 2022A1515012623), Science and Technology Special Fund of Guangdong Province of China (190829105556145), and Strategic and Special Fund for Science and Technology Innovation of Guangdong Province of China (180918114960704).

## Conflict of interest

The authors declare that the research was conducted in the absence of any commercial or financial relationships that could be construed as a potential conflict of interest.

## Publisher’s note

All claims expressed in this article are solely those of the authors and do not necessarily represent those of their affiliated organizations, or those of the publisher, the editors and the reviewers. Any product that may be evaluated in this article, or claim that may be made by its manufacturer, is not guaranteed or endorsed by the publisher.

## Abbreviations

HBM: Health belief model
COVID-19: Coronavirus 2019
BMI: Body mass index
RMB: Renminbi
ORu: Univariate odds ratio
CI: Confidence interval
ORa: adjusted odds ratio

## References

[1] Crook H Raza S Nowell J Young M Edison P. Long covid-mechanisms, risk factors, and management. BMJ. (2021) 374:n1648. doi: 10.1136/bmj.n1648 34312178

[2] Wang B Li R Lu Z Huang Y. Does comorbidity increase the risk of patients with COVID-19: evidence from meta-analysis. Aging (Albany NY). (2020) 12:6049–57. doi: 10.18632/aging.103000 32267833 PMC7185114

[3] Yang J Zheng Y Gou X Pu K Chen Z Guo Q. Prevalence of comorbidities and its effects in patients infected with SARS-CoV-2: a systematic review and meta-analysis. Int J Infect Dis. (2020) 94:91–5. doi: 10.1016/j.ijid.2020.03.017, PMID: 32173574 PMC7194638

[4] Hajj Hussein I Chams N Chams S el Sayegh S Badran R Raad M. Vaccines through centuries: major cornerstones of Global Health. Front Public Health. (2015) 3:269. doi: 10.3389/fpubh.2015.00269, PMID: 26636066 PMC4659912

[5] Watson OJ Barnsley G Toor J Hogan AB Winskill P Ghani AC. Global impact of the first year of COVID-19 vaccination: a mathematical modelling study. Lancet Infect Dis. (2022) 22:1293–302. doi: 10.1016/S1473-3099(22)00320-6 35753318 PMC9225255

[6] Venkatesulu BP Chandrasekar VT Girdhar P Advani P Sharma A Elumalai T. A systematic review and meta-analysis of cancer patients affected by a novel coronavirus. JNCI Cancer Spectr. (2021) 5:a102. doi: 10.1093/jncics/pkaa102, PMID: 33875976 PMC7928783

[7] Dai M Liu D Liu M Zhou F Li G Chen Z. Patients with cancer appear more vulnerable to SARS-CoV-2: a multicenter study during the COVID-19 outbreak. Cancer Discov. (2020) 10:783–91. doi: 10.1158/2159-8290.CD-20-0422, PMID: 32345594 PMC7309152

[8] Kuderer NM Choueiri TK Shah DP Shyr Y Rubinstein SM Rivera DR. Clinical impact of COVID-19 on patients with cancer (CCC19): a cohort study. Lancet. (2020) 395:1907–18. doi: 10.1016/S0140-6736(20)31187-9, PMID: 32473681 PMC7255743

[9] Giannakoulis VG Papoutsi E Siempos II. Effect of cancer on clinical outcomes of patients with COVID-19: a meta-analysis of patient data. JCO Glob Oncol. (2020) 6:799–808. doi: 10.1200/GO.20.00225 32511066 PMC7328119

[10] Liang W Guan W Chen R Wang W Li J Xu K. Cancer patients in SARS-CoV-2 infection: a nationwide analysis in China. Lancet Oncol. (2020) 21:335–7. doi: 10.1016/S1470-2045(20)30096-6, PMID: 32066541 PMC7159000

[11] Ma Y Ren J Zheng Y Cai D Li S Li Y. Chinese parents' willingness to vaccinate their children against COVID-19: a systematic review and meta-analysis. Front Public Health. (2022) 10:1087295. doi: 10.3389/fpubh.2022.1087295 36590001 PMC9798204

[12] Almalki OS Alfayez OM Al YM Asiri YA Almohammed OA. Parents' hesitancy to vaccinate their 5-11-year-old children against COVID-19 in Saudi Arabia: predictors from the health belief model. Front Public Health. (2022) 10:842862. doi: 10.3389/fpubh.2022.842862 35433579 PMC9005777

[13] Forster M Wuerstlein R Koenig A Amann N Beyer S Kaltofen T. COVID-19 vaccination in patients with breast cancer and gynecological malignancies: a German perspective. Breast. (2021) 60:214–22. doi: 10.1016/j.breast.2021.10.012, PMID: 34736092 PMC8555340

[14] Thomas SJ Perez JL Lockhart SP Hariharan S Kitchin N Bailey R. Efficacy and safety of the BNT162b2 mRNA COVID-19 vaccine in participants with a history of cancer: subgroup analysis of a global phase 3 randomized clinical trial. Vaccine. (2022) 40:1483–92. doi: 10.1016/j.vaccine.2021.12.046, PMID: 35131133 PMC8702495

[15] Qi X Wang J Zhang Q Ai J Liu C Li Q. Safety and immunogenicity of COVID-19 vaccination in patients with hepatocellular carcinoma (CHESS-NMCID 2101): a multicenter prospective study. J Med Virol. (2022) 94:5553–9. doi: 10.1002/jmv.27992, PMID: 35811309 PMC9350086

[16] Wu JT la J Branch-Elliman W Huhmann LB Han SS Parmigiani G. Association of COVID-19 vaccination with SARS-CoV-2 infection in patients with cancer: a US Nationwide veterans affairs study. JAMA Oncol. (2022) 8:281–6. doi: 10.1001/jamaoncol.2021.5771, PMID: 34854921 PMC8640949

[17] Iacono D Cerbone L Palombi L Cavalieri E Sperduti I Cocchiara RA. Serological response to COVID-19 vaccination in patients with cancer older than 80 years. J Geriatr Oncol. (2021) 12:1253–5. doi: 10.1016/j.jgo.2021.06.002, PMID: 34175246 PMC8192957

[18] Mislang AR Soto-Perez-de-Celis E Russo C Colloca G Williams GR O'Hanlon S. The SIOG COVID-19 working group recommendations on the rollout of COVID-19 vaccines among older adults with cancer. J Geriatr Oncol. (2021) 12:848–50. doi: 10.1016/j.jgo.2021.03.003, PMID: 33715995 PMC7934668

[19] Garassino MC Vyas M de Vries E Kanesvaran R Giuliani R Peters S. The ESMO call to action on COVID-19 vaccinations and patients with cancer: vaccinate. Monitor Educate Ann Oncol. (2021) 32:579–81. doi: 10.1016/j.annonc.2021.01.068 33582237 PMC7879154

[20] Loh KP Soto-Perez-de-Celis E Mislang AR Chan WL Battisti N. COVID-19 vaccines in older adults with cancer: a Young International Society of Geriatric Oncology perspective. Lancet Healthy Longev. (2021) 2:e240–2. doi: 10.1016/S2666-7568(21)00060-X 33977283 PMC8102034

[21] Desai A Gainor JF Hegde A Schram AM Curigliano G Pal S. COVID-19 vaccine guidance for patients with cancer participating in oncology clinical trials. Nat Rev Clin Oncol. (2021) 18:313–9. doi: 10.1038/s41571-021-00487-z, PMID: 33723371 PMC7957448

[22] Ribas A Sengupta R Locke T Zaidi SK Campbell KM Carethers JM. Priority COVID-19 vaccination for patients with cancer while vaccine supply is limited. Cancer Discov. (2021) 11:233–6. doi: 10.1158/2159-8290.CD-20-1817, PMID: 33355178 PMC8053003

[23] American Society of Clinical Oncology. COVID-19 vaccines & patients with cancer. (2021). Available at: https://www.asco.orgasco-coronavirus-resources/covid-19-vaccines-patients-cancer. (Accessed May 12, 2022).

[24] National Comprehensive Cancer Network. Recommendations of the NCCN COVID-19 vaccination advisorycommittee version 2.0. (2021). Available at: (https://www.nccn.org/covid-19/).

[25] Chinese Anti-Cancer Association Tumor Supportive Therapy Professional Committee, Chinese Anti-Cancer Association Cancer Clinical Chemotherapy Professional Committee. Chinese expert consensus on issues related to the protection, treatment and management of patients with solid tumors during COVID-19 (2022 edition). Zhonghua Zhong Liu Za Zhi. (2022) 44:1083–90. doi: 10.3760/cma.j.cn112152-20220505-00309 36319453

[26] Heyne S Esser P Werner A Lehmann-Laue A Mehnert-Theuerkauf A. Attitudes toward a COVID-19 vaccine and vaccination status in cancer patients: a cross-sectional survey. J Cancer Res Clin Oncol. (2022) 148:1363–74. doi: 10.1007/s00432-022-03961-y 35218401 PMC8881553

[27] Suzuki H Akiyama T Ueda N Matsumura S Mori M Namiki M. COVID-19 vaccination in patients with cancer. Cancers (Basel). (2022) 14:2556. doi: 10.3390/cancers14102556, PMID: 35626162 PMC9139318

[28] Powis M Sutradhar R Patrikar A Cheung M Gong I Vijenthira A. Factors associated with timely COVID-19 vaccination in a population-based cohort of patients with cancer. J Natl Cancer Inst. (2023) 115:146–54. doi: 10.1093/jnci/djac204, PMID: 36321960 PMC9905967

[29] Marijanovic I Kraljevic M Buhovac T Sokolovic E. Acceptance of COVID-19 vaccination and its associated factors among cancer patients attending the oncology Clinic of University Clinical Hospital Mostar, Bosnia and Herzegovina: a cross-sectional study. Med Sci Monit. (2021) 27:e932788. doi: 10.12659/MSM.932788 34772907 PMC8596742

[30] Mejri N Berrazega Y Ouertani E Rachdi H Bohli M Kochbati L. Understanding COVID-19 vaccine hesitancy and resistance: another challenge in cancer patients. Support Care Cancer. (2022) 30:289–93. doi: 10.1007/s00520-021-06419-y, PMID: 34279721 PMC8286987

[31] Villarreal-Garza C Vaca-Cartagena BF Becerril-Gaitan A Ferrigno AS Mesa-Chavez F Platas A. Attitudes and factors associated with COVID-19 vaccine hesitancy among patients with breast cancer. JAMA Oncol. (2021) 7:1242–4. doi: 10.1001/jamaoncol.2021.1962, PMID: 34110371 PMC8193544

[32] Chun JY Kim SI Park EY Park SY Koh SJ Cha Y. Cancer Patients' willingness to take COVID-19 vaccination: a Nationwide multicenter survey in Korea. Cancers (Basel). (2021) 13:3883. doi: 10.3390/cancers13153883, PMID: 34359783 PMC8345425

[33] Peng X Gao P Wang Q Wu HG Yan YL Xia Y. Prevalence and impact factors of covid-19 vaccination hesitancy among breast cancer survivors: a multicenter cross-sectional study in China. Front Med (Lausanne). (2021) 8:741204. doi: 10.3389/fmed.2021.741204, PMID: 34805207 PMC8595240

[34] Hong J Xu XW Yang J Zheng J Dai SM Zhou J. Knowledge about, attitude and acceptance towards, and predictors of intention to receive the COVID-19 vaccine among cancer patients in eastern China: a cross-sectional survey. J Integr Med. (2022) 20:34–44. doi: 10.1016/j.joim.2021.10.004, PMID: 34774463 PMC8559872

[35] Zhuang W Zhang J Wei P Lan Z Chen R Zeng C. Misconception contributed to COVID-19 vaccine hesitancy in patients with lung cancer or ground-glass opacity: a cross-sectional study of 324 Chinese patients. Hum Vaccin Immunother. (2021) 17:5016–23. doi: 10.1080/21645515.2021.1992212, PMID: 34715002 PMC8903957

[36] Liu W Wu Y Yang R Chen R Huang Y Zhao X. COVID-19 vaccination status and hesitancy among breast cancer patients after two years of pandemic: a cross-sectional survey. Vaccines (Basel). (2022) 10:1530. doi: 10.3390/vaccines10091530, PMID: 36146608 PMC9503096

[37] Chan WL Ho YT Wong CK Choi HC Lam KO Yuen KK. Acceptance of COVID-19 vaccination in cancer patients in Hong Kong: approaches to improve the vaccination rate. Vaccines (Basel). (2021) 9:792. doi: 10.3390/vaccines9070792, PMID: 34358208 PMC8310340

[38] Wang Y Zhang L Chen S Lan X Song M Su R. Hesitancy to receive the booster doses of COVID-19 vaccine among cancer patients in China: a multicenter cross-sectional survey - four PLADs, China, 2022. China CDC Wkly. (2023) 5:223–8. doi: 10.46234/ccdcw2023.041, PMID: 37006443 PMC10061813

[39] Zhang L Yang J Su R du X Wang Y Chen S. Concerns related to the interactions between COVID-19 vaccination and cancer/cancer treatment were barriers to complete primary vaccination series among Chinese cancer patients: a multicentre cross-sectional survey. Hum Vaccin Immunother. (2023) 19:2222648. doi: 10.1080/21645515.2023.2222648, PMID: 37314490 PMC10332204

[40] Barrière J Gal J Hoch B Cassuto O Leysalle A Chamorey E. Acceptance of SARS-CoV-2 vaccination among French patients with cancer: a cross-sectional survey. Ann Oncol. (2021) 32:673–4. doi: 10.1016/j.annonc.2021.01.066, PMID: 33529740 PMC7846886

[41] Prabani K Weerasekara I Damayanthi H. COVID-19 vaccine acceptance and hesitancy among patients with cancer: a systematic review and meta-analysis. Public Health. (2022) 212:66–75. doi: 10.1016/j.puhe.2022.09.001 36244261 PMC9452406

[42] di Noia V Renna D Barberi V di Civita M Riva F Costantini G. The first report on coronavirus disease 2019 (COVID-19) vaccine refusal by patients with solid cancer in Italy: early data from a single-institute survey. Eur J Cancer. (2021) 153:260–4. doi: 10.1016/j.ejca.2021.05.006, PMID: 34183225 PMC8149194

[43] Szilagyi PG Thomas K Shah MD Vizueta N Cui Y Vangala S. Likelihood of COVID-19 vaccination by subgroups across the US: post-election trends and disparities. Hum Vaccin Immunother. (2021) 17:3262–7. doi: 10.1080/21645515.2021.1929695, PMID: 34170793 PMC8437533

[44] Kelkar AH Blake JA Cherabuddi K Cornett H McKee BL Cogle CR. Vaccine enthusiasm and hesitancy in cancer patients and the impact of a webinar. Healthcare (Basel). (2021) 9:351. doi: 10.3390/healthcare9030351 33808758 PMC8003419

[45] Walsh JC Comar M Folan J Williams S Kola-Palmer S. The psychological and behavioural correlates of COVID-19 vaccine hesitancy and resistance in Ireland and the UK. Acta Psychol. (2022) 225:103550. doi: 10.1016/j.actpsy.2022.103550 PMC8882412 35259642

[46] Moscardino U Musso P Inguglia C Ceccon C Miconi D Rousseau C. Sociodemographic and psychological correlates of COVID-19 vaccine hesitancy and resistance in the young adult population in Italy. Vaccine. (2022) 40:2379–87. doi: 10.1016/j.vaccine.2022.03.018 35305828 PMC8920409

[47] Paul E Steptoe A Fancourt D. Attitudes towards vaccines and intention to vaccinate against COVID-19: implications for public health communications. Lancet Reg Health Eur. (2021) 1:100012. doi: 10.1016/j.lanepe.2020.100012 33954296 PMC7834475

[48] Glanz K Bishop DB. The role of behavioral science theory in development and implementation of public health interventions. Annu Rev Public Health. (2010) 31:399–418. doi: 10.1146/annurev.publhealth.012809.103604 20070207

[49] Getachew T Lami M Eyeberu A Balis B Debella A Eshetu B. Acceptance of COVID-19 vaccine and associated factors among health care workers at public hospitals in eastern Ethiopia using the health belief model. Front Public Health. (2022) 10:957721. doi: 10.3389/fpubh.2022.957721, PMID: 36438218 PMC9683340

[50] Youssef D Abou-Abbas L Berry A Youssef J Hassan H. Determinants of acceptance of coronavirus disease-2019 (COVID-19) vaccine among Lebanese health care workers using health belief model. PLoS One. (2022) 17:e264128. doi: 10.1371/journal.pone.0264128 PMC8863223 35192664

[51] Tao L Wang R Han N Liu J Yuan C Deng L. Acceptance of a COVID-19 vaccine and associated factors among pregnant women in China: a multi-center cross-sectional study based on health belief model. Hum Vaccin Immunother. (2021) 17:2378–88. doi: 10.1080/21645515.2021.1892432, PMID: 33989109 PMC8475603

[52] Qin C Yan W du M Liu Q Tao L Liu M. Acceptance of the COVID-19 vaccine booster dose and associated factors among the elderly in China based on the health belief model (HBM): a national cross-sectional study. Front Public Health. (2022) 10:986916. doi: 10.3389/fpubh.2022.986916, PMID: 36589991 PMC9797829

[53] Lin Y Hu Z Zhao Q Alias H Danaee M Wong LP. Understanding COVID-19 vaccine demand and hesitancy: a nationwide online survey in China. PLoS Negl Trop Dis. (2020) 14:e8961. doi: 10.1371/journal.pntd.0008961 PMC7775119 33332359

[54] Chen H Li X Gao J Liu X Mao Y Wang R. Health belief model perspective on the control of COVID-19 vaccine hesitancy and the promotion of vaccination in China: web-based cross-sectional study. J Med Internet Res. (2021) 23:e29329. doi: 10.2196/29329, PMID: 34280115 PMC8425399

[55] Mirzaei A Kazembeigi F Kakaei H Jalilian M Mazloomi S Nourmoradi H. Application of health belief model to predict COVID-19-preventive behaviors among a sample of Iranian adult population. J Educ Health Promot. (2021) 10:69. doi: 10.4103/jehp.jehp_747_20 34084816 PMC8057168

[56] Zhu W Zou H Song Y Ren L Xu Y. Understanding the impact process of vaccine adoption for COVID-19. Hum Vaccin Immunother. (2022) 18:2099166. doi: 10.1080/21645515.2022.2099166 35905384 PMC9746427

[57] Schaefer ZK Hoffman MA. Beliefs and attitudes regarding human papillomavirus vaccination among college-age women. J Health Psychol. (2013) 18:1360–70. doi: 10.1177/1359105312462432 23188917

[58] Shahrabani S Benzion U. How experience shapes health beliefs: the case of influenza vaccination. Health Educ Behav. (2012) 39:612–9. doi: 10.1177/1090198111427411 22287574

[59] Rani M Mohamed NA Solehan HM Ithnin M Ariffien AR Isahak I. Assessment of acceptability of the COVID-19 vaccine based on the health belief model among Malaysians-a qualitative approach. PLoS One. (2022) 17:e269059. doi: 10.1371/journal.pone.0269059 PMC9197030 35700197

[60] Xu J Chen S Wang Y Duan L Li J Shan Y. Prevalence and determinants of COVID-19 vaccination uptake were different between Chinese diabetic inpatients with and without chronic complications: a cross-sectional survey. Vaccines (Basel). (2022) 10:994. doi: 10.3390/vaccines10070994, PMID: 35891159 PMC9317053

[61] Rodriguez M Lopez-Cepero A Ortiz-Martinez AP Fernandez-Repollet E Perez CM. Influence of health beliefs on COVID-19 vaccination among individuals with cancer and other comorbidities in Puerto Rico. Vaccines (Basel). (2021) 9:994. doi: 10.3390/vaccines9090994 34579231 PMC8473277

[62] Yu Y Lau J She R Chen X Li L Li L. Prevalence and associated factors of intention of COVID-19 vaccination among healthcare workers in China: application of the health belief model. Hum Vaccin Immunother. (2021) 17:2894–902. doi: 10.1080/21645515.2021.1909327, PMID: 33877955 PMC8381834

[63] Moore JX Gilbert KL Lively KL Laurent C Chawla R Li C. Correlates of COVID-19 vaccine hesitancy among a community sample of African Americans living in the southern United States. Vaccines (Basel). (2021) 9:879. doi: 10.3390/vaccines9080879, PMID: 34452004 PMC8402307

[64] The coverage rate of the first dose of COVID-19 vaccine in Shantou is nearly 95 percent. (2022). Available at: https://www.shantou.gov.cn/ (Accessed March 17, 2023).

[65] Ugas MA Avery L Wang Y Berlin A Giuliani ME Krzyzanowska M. COVID-19 and cancer patients in the second year of the pandemic: investigating treatment impact, information sources, and COVID-19-related knowledge, attitudes and practices. CO. (2022) 29:8917–36. doi: 10.3390/curroncol29110701, PMID: 36421354 PMC9689213

[66] Overheu O Lendowski S Quast DR Marheinecke CS Kourti E Lugnier C. Attitude towards and perception of individual safety after SARS-CoV-2 vaccination among German cancer patients. J Cancer Res Clin Oncol. (2023) 149:1985–92. doi: 10.1007/s00432-022-04099-7, PMID: 35731276 PMC9215322

[67] Brodziak A Sigorski D Osmola M Wilk M Gawlik-Urban A Kiszka J. Attitudes of patients with cancer towards vaccinations-results of online survey with special focus on the vaccination against COVID-19. Vaccines (Basel). (2021) 9:411. doi: 10.3390/vaccines9050411, PMID: 33919048 PMC8142983

[68] Contraindications of vaccination in COVID-19. (2022). Available at: http://wsjkw.gd.gov.cn (Accessed March 20, 2023).

[69] Ionescu TC Fetecau BI Giurgiuca A Tudose C. Acceptance and factors influencing acceptance of COVID-19 vaccine in a Romanian population. J Pers Med. (2022) 12:452. doi: 10.3390/jpm12030452 35330452 PMC8955399

[70] Iscan G Cetin B Kilic F Kalayci H Kalayci A Iscan SC. Investigation of anxiety sensitivity levels of cancer patients in terms of COVID-19 vaccine: a cross-sectional study. Support Care Cancer. (2022) 30:4139–47. doi: 10.1007/s00520-021-06750-4 35067730 PMC8784216

[71] Nguyen M Bain N Grech L Choi T Harris S Chau H. COVID-19 vaccination rates, intent, and hesitancy in patients with solid organ and blood cancers: a multicenter study. Asia Pac J Clin Oncol. (2022) 18:570–7. doi: 10.1111/ajco.13754, PMID: 35043559

[72] Shacham Abulafia A Shemesh S Rosenmann L Berger T Leader A Sharf G. Health-related quality of life in patients with chronic myeloid leukemia treated with first- versus second-generation tyrosine kinase inhibitors. J Clin Med. (2020) 9:3417. doi: 10.3390/jcm9113417, PMID: 33113857 PMC7692789

[73] Shmueli L. Predicting intention to receive COVID-19 vaccine among the general population using the health belief model and the theory of planned behavior model. BMC Public Health. (2021) 21:804. doi: 10.1186/s12889-021-10816-7 33902501 PMC8075011

[74] Magee L Knights F Mckechnie D Al-Bedaery R Razai MS. Facilitators and barriers to COVID-19 vaccination uptake among ethnic minorities: a qualitative study in primary care. PLoS One. (2022) 17:e270504. doi: 10.1371/journal.pone.0270504 PMC9269906 35802738

[75] Kricorian K Civen R Equils O. COVID-19 vaccine hesitancy: misinformation and perceptions of vaccine safety. Hum Vaccin Immunother. (2022) 18:1950504. doi: 10.1080/21645515.2021.1950504 34325612 PMC8920251

[76] Olusanya OA Bednarczyk RA Davis RL Shaban-Nejad A. Addressing parental vaccine hesitancy and other barriers to childhood/adolescent vaccination uptake during the coronavirus (COVID-19) pandemic. Front Immunol. (2021) 12:663074. doi: 10.3389/fimmu.2021.663074 33815424 PMC8012526

[77] Rocque GB Caston NE Andrews C England R Williams CP Azuero A. Vaccine hesitancy versus vaccine behavior in patients with chronic illness. J Health Care Poor Underserved. (2022) 33:2007–31. doi: 10.1353/hpu.2022.0150, PMID: 36341675

[78] Bhagianadh D Arora K. COVID-19 vaccine hesitancy among community-dwelling older adults: the role of information sources. J Appl Gerontol. (2022) 41:4–11. doi: 10.1177/07334648211037507 34365856

[79] Chen X Giles J Yao Y Yip W Meng Q Berkman L. The path to healthy ageing in China: a Peking University-Lancet Commission. Lancet. (2022) 400:1967–2006. doi: 10.1016/S0140-6736(22)01546-X, PMID: 36423650 PMC9801271

[80] Duan L Wang Y Dong H Song C Zheng J Li J. The COVID-19 vaccination behavior and correlates in diabetic patients: a health belief model theory-based cross-sectional study in China, 2021. Vaccines (Basel). (2022) 10:659. doi: 10.3390/vaccines10050659, PMID: 35632415 PMC9148061

[81] Epidemic situation in COVID-19, Guangdong Province. (2022). Available at: (https://www.shantou.gov.cn).

[82] Cai Z Hu W Zheng S Wen X Wu K. Cognition and behavior of COVID-19 vaccination based on the health belief model: a cross-sectional study. Vaccines (Basel). (2022) 10:544. doi: 10.3390/vaccines10040544 35455293 PMC9030847

[83] Khubchandani J Bustos E Chowdhury S Biswas N Keller T. COVID-19 vaccine refusal among nurses worldwide: review of trends and predictors. Vaccines (Basel). (2022) 10:230. doi: 10.3390/vaccines10020230 35214687 PMC8876951

