# Supplementary material for: Prevalence and factors of COVID-19 vaccine refusal among solid cancer patients in China: an application of the health belief model
Source: Front Public Health. 2023 Aug 3;11:1236376. doi: 10.3389/fpubh.2023.1236376 (PMC10435902; doi:10.3389/fpubh.2023.1236376)
Supplement: Supplementary file 1 [file Data_Sheet_1.pdf]

Supplementary Table 1. Descriptions of scale, indicator, items, and reliability coefficients

Scales and range	Items	Cronbach's α / Proportion (%)
Perceived Susceptibility of COVID-19 Scale<br><br>(range = 0 to 8)	1. You would not contract COVID-19 in the next year. (score reversed)<br>2. According to the current situation in China, the risk of COVID-19 infection in the general population is very low. (score reversed)	0.73
Perceived Severity of COVID-19 Scale<br><br>(range = 0 to 12)	1. In the case of COVID-19 infection, cancer patients would be more likely than the general population to have severe complications.<br>2. In the case of COVID-19 infection, cancer patients' tumor conditions (e.g., recurrence or treatment) would be aggravated by COVID-19.<br>3. In the case of COVID-19 infection, cancer patients would have increased risk of contracting other infectious diseases.	0.89
Perceived Benefits of COVID-19 Vaccination Scale<br><br>(range = 0 to 12)	1. COVID-19 vaccination could effectively protect me from COVID-19 infection.<br>2. COVID-19 vaccination could effectively reduce the disease severity and mortality related to COVID-19 infection.<br>3. Taking up COVID-19 vaccination could make my family members and me psychologically relieved.	0.81
Perceived Barriers of COVID-19 Vaccination Scale<br><br>(range = 0 to 12)	1. There are practical difficulties (e.g., cancer condition) that make me unable to take up COVID-19 vaccination.<br>2. Cancer patients would have more or worse side effects related to COVID-19 vaccination than the general population.<br>3. My health condition is too weak to take up COVID-19 vaccination.	0.81
Self-Efficacy of COVID-19 Vaccination Scale (range = 0 to 4)	1. If you would like to take up COVID-19 vaccination, are you confident to do so?	-
Cue to Action of COVID-19 Vaccination Indicator	1. My family suggested that I go to take up COVID-19 vaccination.	242 (21.7)
	2. The doctor or nurse suggested me to take up COVID-19 vaccination.	293 (26.2)
	3. Good friends suggested me take up COVID-19 vaccination.	130 (11.6)
(a count variable; range: 0 to 4)	4. Community/village committee members suggested me to take up COVID-19 vaccination.	235 (21.0)

Supplementary Table 2. Adjusted associations between the HBM Variables and COVID-19 Vaccination Inclination ('Vaccine non-refusal' versus ever-vaccination).

HBM Variables	'Vaccine non-refusal' versus Ever-vaccination
	ORa (95% CI)
Perceived Susceptibility	1.01 (0.80, 1.28)
Perceived Severity	1.23 (0.97, 1.55)
Perceived Benefits	0.31 (0.23, 0.43) ***
Perceived Barriers	13.62 (9.19, 20.19) ***
Cue to Action (number of types of suggestion)	
2-4	0.06 (0.03, 0.1) ***
1	0.13 (0.08, 0.21) ***
0	Ref
Self-Efficacy	0.33 (0.27, 0.4) ***

Note: These models adjusted for age, current marital status, BMI, marital status, education, job status, number of family members, Income, cancer type, time since cancer diagnosis, current treatment status. BMI = Body Mass Index; RMB = Renminbi; HBM = Health Belief Model; ORa = adjusted odds ratio; CI = Confidence interval; *, $p < 0.05$; ***, $p < 0.001$.
